# A focus on CKD reporting and inappropriate prescribing among older patients discharged from geriatric and nephrology units throughout Italy: A nationwide multicenter retrospective cross-sectional study

**DOI:** 10.3389/fphar.2022.996042

**Published:** 2022-10-14

**Authors:** Filippo Aucella, Andrea Corsonello, Luca Soraci, Paolo Fabbietti, Michele Antonio Prencipe, Giuseppe Gatta, Fabrizia Lattanzio, Livio Cortese, Maria Rosaria Pagnotta, Raffaele Antonelli Incalzi

**Affiliations:** ^1^ SC di Nefrologia e Dialisi, IRCCS “Casa Sollievo della Sofferenza”, San Giovanni Rotondo, Foggia, Italy; ^2^ Unit of Geriatric Medicine, IRCCS INRCA, Cosenza, Italy; ^3^ Laboratory of Pharmacoepidemiology and Biostatistics, IRCCS INRCA, Ancona, Italy; ^4^ Scientific Direction, IRCCS INRCA, Ancona, Italy; ^5^ Department of Geriatrics, Campus Biomedico, Rome, Italy

**Keywords:** inappropriate prescribing, CKD, real-world scenario, acute care units, hospital setting, older patients

## Abstract

Older hospitalized patients with chronic kidney disease (CKD) are part of the geriatric population with a substantial risk of potentially inappropriate medication (PIM) use. The high rates of multimorbidity and polypharmacy, along with the progressive decline of eGFR, contribute to increasing the risk of drug–drug and drug–disease interactions, overdosing, and adverse drug reactions (ADRs). In this multicenter cross-sectional study, we aimed to evaluate the prevalence of CKD under-reporting and PIMs among older patients discharged from acute geriatric and nephrology units throughout Italy. Renal function was determined by estimated glomerular filtration rate (eGFR) through the Berlin Initiative Study (BIS) equation; the prevalence of PIMs was calculated by revising drug prescriptions at discharge according to STOPP criteria, Beers criteria, and summaries of product characteristics (smPCs). A descriptive analysis was performed to compare the clinical and pharmacological characteristics of patients in the two distinct settings; univariate and multivariate logistic regression models were performed to explore factors associated with CKD under-reporting in the discharge report forms and PIM prevalence. Overall, the study population consisted of 2,057 patients, aged 83 (77–89) years, more commonly women, with a median of seven (5–10) drugs prescribed at discharge. CKD under-reporting was present in 50.8% of the study population, with higher rates in geriatric vs. nephrology units (71.1% vs. 10.2%, *p* < 0.001). 18.5% of the study population was discharged with at least one renally inappropriate medication; factors associated with at least one contraindicated drug at discharge were the number of drugs (PR 1.09, 95% CI 1.14–1.19); atrial fibrillation (PR 1.35, 95% CI 1.01–1.81); diabetes (PR 1.61, 95% CI 1.21–2.13); being hospitalized in nephrology units (PR 1.62, 95% CI 1.14–2.31), CKD stage 3b (PR 2.35, 95% CI 1.34–4.13), and stage 4–5 (PR 14.01, 95% CI 7.36–26.72). Conversely, CKD under-reporting was not associated with the outcome. In summary, CKD under-reporting and inappropriate medication use were common in older patients discharged from hospital; the relatively high number of PIMs in both nephrology and geriatric settings underlines the need to improve appropriate prescribing during hospital stay and to decrease the risk of ADRs and side effects in this highly vulnerable population.

## 1 Introduction

Chronic kidney disease (CKD) and polypharmacy have been recognized as major public health threats worldwide (Cockwell and Fisher, 2020; Global, 2020), with an increasing prevalence and burden across distinct care settings and populations (Schmidt et al., 2019; Shouqair et al., 2021); older patients are particularly prone to CKD development and progression, as a result of progressive age-induced physiological changes and increased multimorbidity and exposure to chronically high blood pressure and glycemic levels (Kazancioğlu, 2013; Denic et al., 2016). Age-related CKD burden is fueled by risks of polypharmacy and drug toxicity; indeed, older patients with CKD often present with multimorbidity, with subsequent increased use of multiple medications (Schmidt et al., 2019). Moreover, CKD patients often need medications to limit the deterioration of kidney function and manage CKD complications.

Given that most drugs are renally excreted, decrease in GFR alters the pharmacokinetic and pharmacodynamic properties of medications (Lea-Henry et al., 2018); as a result, polypharmacy could make drug management more difficult and lead to adverse drug events (ADEs), drug–drug interactions, and potentially inappropriate medication (PIM) use (Barbieri et al., 2020); furthermore, inappropriate prescription of renally excreted medications may lead to further progression and worsening of kidney function over time, which ulteriorly increases risks of PIM use in this population (Schmidt et al., 2019). Indeed, several medications, including antidiabetics, beta-blockers, non-steroidal anti-inflammatory drugs (NSAIDs), and renin–angiotensin–aldosterone system (RAAS) inhibitors, have shown nephrotoxic potential (Okoro and Farate, 2019). In this regard, in 2017, the World Health Organization (WHO) highlighted the need to address unsafe medication regimens and errors, which are often associated with an increased individual risk/benefit ratio (Okpechi et al., 2021). Adherence to validated criteria of appropriate prescriptions, such as the screening tool to alert to right treatment/screening tool of older people’s prescriptions (START/STOPP) (O’Mahony, 2020); and American Geriatric Society (AGS) Beers criteria (by the 2019 American Geriatrics Society Beers Criteria^®^ Update Expert Panel, 2019), may serve as a starting point to revise pharmacological regimens, especially during hospital stay; however, the silent course of CKD may delay the diagnosis of the disease and provision of appropriate treatment options to avoid polypharmacy burden (Fiseha et al., 2014; Carriazo et al., 2021).

Previous studies have highlighted the common use of PIMs and renally inappropriate medications among hospitalized older patients with CKD (Tesfaye et al., 2019; Chahine, 2020; Sheikh Rezaei et al., 2021), suggesting a lack of appropriate assessments of medication regimens as kidney function deteriorates; however, no study has previously reported on differences in CKD diagnosis and PIM use among geriatric and nephrological acute care settings. The present study, performed in wards of Geriatrics and Nephrology, aims to assess 1) the prevalence of CKD under-reporting at hospital discharge; 2) the prevalence of inappropriate prescription/dosing of renally excreted drugs at hospital discharge; and 3) factors associated with higher rates of PIMs and CKD under-reporting in the overall population and specific settings.

## 2 Materials and methods

### 2.1 Study design and data collection

The present study uses data from the Italian Society of Nephrology-Italian Society of Gerontology and Geriatrics (SIN-SIGG) study, an observational, retrospective, cross-sectional study cooperatively funded by the Italian Society of Nephrology (SIN) and the Italian Society of Gerontology and Geriatrics (SIGG); this study aimed at investigating the prevalence of appropriate CKD reporting and medication prescribing/dosing in patients aged 65 years or more consecutively discharged from twenty-four geriatric and fifteen nephrology units in Italy within the first half of 2018. The study design complies with the Declaration of Helsinki and Good Clinical Practice Guidelines. The study protocol was approved by the ethics committees at all participating institutions.

All records and hospital discharges of patients aged 65 years or more admitted to the participating geriatrics and nephrology units within the first half of 2018 have been reviewed without any other specific inclusion criteria. In case of multiple hospitalizations for the same patient during the identified observation period, only the first hospitalization has been included. Patients undergoing dialysis, those discharged within 24 h after hospital admission, those who died during hospital stay, and those with missing data for serum creatinine and prescribed drugs were excluded from the study.

The characteristics of the study population were retrospectively retrieved by each medical record in participating institutions and included demographic data (age, sex, weight, height, and body mass index), systolic and diastolic blood pressure, heart rate, number and type of diseases, and medications prescribed. All diagnoses were coded according to the International Classification of Diseases Ninth Edition (ICD-9 Clinical Modification) system (Iezzoni, 1990), and the prescribed drugs at admission and discharge were assessed by the Anatomic Therapeutic Chemical (ATC) Classification System (Miller and Britt, 1995). Polypharmacy was defined as taking five or more medications (Gnjidic et al., 2012).

#### 2.1.1 eGFR assessment

The estimated glomerular filtration rate (eGFR) was calculated at admission and discharge by using the Berlin-Initiative-Study 1 (BIS1) equation, which was specifically developed for a population older than 70 years (Schaeffner et al., 2012):

$$BIS1=3736\times S_{Cr}^{-0.87}{\times age}^{-0.95}\;\left[\times 0.82\;if\;female\right].$$

According to the eGFR, patients were classified into stages G1–G2 (≥60 ml/min/1.73 m^2^), stage G3a (eGFR 45–59 ml/min/1.73 m^2^), stage G3b (eGFR 30–44 ml/min/1.73 m^2^), and stage G4-5 (eGFR < 30 ml/min/1.73 m^2^). The diagnosis of CKD was based on having an eGFR lower than 60 ml/min/1.73 m^2^ both at admission and discharge, as well as a personal history of CKD. The prevalence of CKD diagnosis at discharge was assessed by reviewing ICD-9 CM codes 403, 404, and 585 on the hospital discharge forms. The prevalence of under-reporting of CKD was calculated by comparing the reported prevalence of CKD at hospital discharge to that of CKD diagnosis.

#### 2.1.2 Potentially inappropriate medications

Renally inappropriate medications are referred to as drugs that should be avoided or that undergo dose modification in patients with CKD.

The prevalence of inappropriate prescribing/dosing PIMs at discharge has been assessed by reviewing three different criteria:

1) STOPP and START v2 criteria (O’Mahony et al., 2015): these criteria were originally defined in 2008 to identify medications not appropriate for older adults; the list was updated in 2015 and comprises 114 criteria: 80 STOPP and 34 START. Among them, we identified six STOPP statements related to potentially nephrotoxic drugs, while no START was specifically related to CKD (Supplementary Box S1).
2) 2019 updated American Geriatric Society (AGS) Beers criteria (By the 2019 American Geriatrics Society Beers Criteria^®^ Update Expert Panel, 2019): developed in the United States and last revised in 2019, they contain explicit criteria divided into six tables: table 2 listing “potentially inappropriate medications in older patients apart from the clinical condition,” table 3 listing “medication use in older adults due to drug–disease or drug–syndrome interactions that may exacerbate the disease or syndrome,” table 4 listing “potentially inappropriate medications in older patients considering the clinical condition,” table 5 listing “potentially inappropriate medications—drugs to be used with caution in older adults,” table 6 listing “potentially clinically important drug–drug interactions that should be avoided in older adults,” and table 7 listing “medications that should be avoided or have their dosage reduced with varying levels of kidney function in older adults.” Among them, we selected twenty-four criteria to assess PIMs in CKD (Supplementary Box S1).
3) Evaluation of nephrotoxicity based on checking of the European Medical Agency (EMA) Summary of Product Characteristics (SmPC), available at the time of the study (Raynor et al., 2014). This allowed a detailed analysis of the appropriateness of prescribing and dosing of numerous renal elimination drugs, such as thiazides and antialdosterones; antibiotics for temporary use; and antidiabetics such as sulfonylureas, repaglinides, and DPP-IV inhibitors (Supplementary Box S1).

PIM prevalence was calculated according to the three kinds of criteria in both the overall study population and the two settings separately; subsequently, we created a binary variable to identify patients with at least one STOPP and/or Beers PIM and those with at least one cumulative PIM (STOPP and/or Beers and/or smPCs).

#### 2.1.3 Statistical analysis

A descriptive analysis was first conducted to compare demographic, clinical, pharmacological, and laboratory characteristics of the study population, stratified according to the admission unit (Geriatrics and Nephrology). Continuous and categorical variables were reported as a median with interquartile range (IQR) and as the number and percentage, respectively. The normality of the distribution of continuous variables was assessed by the use of the Kolmogorov–Smirnov test. The Mann–Whitney *U* test and two-tailed chi-squared Pearson’s tests were used to compare continuous and categorical variables between geriatric and nephrology units. Univariate and multivariate logistic regression models were then used to investigate factors associated with CKD under-reporting and PIM in both the study population and in distinct settings. Factors significantly associated with the outcomes in the univariate models were included in a multivariate logistic regression model. Collinearity was verified by Spearman’s rank correlation coefficient and the variance inflation factor (VIF). Prevalence ratios (PRs) with 95% CIs were calculated for each covariate of interest in bivariate (age- and sex-adjusted PR) and multivariate (fully adjusted PR) regression models. Sensitivity analyses were additionally carried out by using the CKD-EPI equation instead of the BIS equation to estimate the eGFR. *P-value* < 0.05 was considered statistically significant. Statistical analysis was performed using R version 4.6.

## 3 Results

### 3.1 Description of the study population

The characteristics of the study population are shown in Table 1. Overall, a total of 2,057 patients aged 83 (77–89), with 987 women (48.0%) and a median 7 (5–9) number of drugs prescribed at discharge were included in the study; of them, 1,497 (72.8%) were hospitalized in Geriatric Units, while the remaining 560 (27.2%) were hospitalized in Nephrology Units. Geriatric patients were older, more commonly women, and with a lower BMI than those of patients hospitalized in Nephrology Units; additionally, geriatric patients had a higher prevalence of cardiovascular diseases, cerebrovascular diseases, COPD, and osteoporosis, while nephrology patients were more commonly affected by hypertension, diabetes, and anemia and were characterized by a higher number of prescribed medications (*p* < 0.001).

**TABLE 1:** Descriptive characteristics of the study population in general and stratified by the type of department.

	Overall population (*n* = 2,057)	Geriatric units (*n* = 1,497)	Nephrology units, (*n* = 560)	*p*
Age, median (IQR)	83 (77–89)	85 (78–90)	79 (73–85)	<0.001
Female sex, *n* (%)	987 (48.0)	753 (50.3)	234 (41.8)	<0.001
BMI, median (IQR)	25.9 (23.0–29.4)	25.5 (22.6–29.1)	26.7 (24.0–30.2)	<0.001
Diagnoses				
Hypertension, *n* (%)	1,615 (78.5)	1,153 (77.0)	462 (82.5)	0.008
Type 2 diabetes mellitus, *n* (%)	705 (34.3)	469 (31.3)	236 (42.1)	<0.001
CHF, *n* (%)	670 (32.6)	568 (37.9)	102 (18.2)	<0.001
Atrial fibrillation, *n* (%)	611 (29.7)	488 (32.6)	123 (22.0)	<0.001
CAD, *n* (%)	346 (16.8)	266 (17.8)	80 (14.3)	0.070
Cerebrovascular disease, *n* (%)	283 (13.8)	235 (15.7)	48 (8.6)	<0.001
COPD, *n* (%)	573 (27.9)	459 (30.7)	114 (20.4)	<0.001
Osteoporosis, *n* (%)	318 (15.5)	256 (17.1)	62 (11.1)	<0.001
Anemia, *n* (%)	1,269 (61.7)	870 (58.1)	399 (71.3)	<0.001
Cancer, *n* (%)	531 (25.8)	379 (25.3)	152 (27.1)	0.432
eGFR, ml/min/m^2^, median (IQR)	43.4 (30.6–53.8)	48.6 (36.1–62.1)	30.2 (22.0–44.2)	<0.001
eGFR, ml/min/m^2^, *n* (%)				<0.001
≥60	482 (23.4)	428 (28.6)	54 (9.6)	
45–59.9	504 (24.5)	424 (28.3)	80 (14.3)	
30–44.9	575 (27.9)	428 (28.6)	147 (26.2)	
≤30	396 (24.1)	217 (14.5)	279 (49.8)	
CKD diagnosis	1,499 (72.9)	998 (66.7)	501 (89.5)	<0.001
CKD in the hospital discharge form	787 (38.2)	317 (21.2)	470 (83.9)	<0.001
CKD under-reporting	761 (50.8)*	710 (71.1)	51 (10.2)	<0.001
Number of medications, median (IQR)	7 (5–9)	7 (5–9)	8 (5–10)	<0.001
Polypharmacy, *n* (%)	1401 (68.1)	993 (66.3)	408 (72.9)	0.006

Acronyms: BMI, body mass index; CAD, coronary artery disease; CKD, chronic kidney disease; COPD, chronic obstructive pulmonary disease; eGFR, estimated glomerular filtration rate; SD, standard deviation; *percentages are calculated out of patients with CKD diagnosis.

The overall prevalence of CKD, defined as having an eGFR < 60 ml/min/1.73 m^2^ both at admission and discharge, as well as a previous history of CKD, was equal to 72.9% of the study population, with a significantly higher prevalence in nephrology units compared to geriatric ones (89.5% vs. 66.7%, *p* < 0.001). Among patients with an eGFR < 60, those hospitalized in nephrology units were predominantly characterized by advanced CKD (G4–5: 55.1%) and moderate stage G3b (29.0%), while geriatric ones were more commonly affected by mild–moderate CKD (G3a: 39.7%; G3b: 40.0%).

### 3.2 Chronic kidney disease under-reporting

After examination of discharge codes, the diagnosis of CKD registered by a physician was retrieved in only 38.2% of the study population (Table 1), with higher values in Nephrology Units than in Geriatric Units (83.9% vs. 21.12%, *p* < 0.001). Consequently, the prevalence of under-reported CKD diagnosis was 50.8%. Geriatric units were characterized by the highest prevalence of CKD under-reported diagnosis (71.1%), while among Nephrology patients, this overall prevalence was 10.2% (*p* < 0.001) of all patients with CKD. Factors associated with CKD under-reporting in logistic regression models are shown in Table 2. Among them, age, female sex, CHF, and being hospitalized in geriatric units were associated with CKD under-reporting in the study population; when considering admission units, age, female sex, CHF, and anemia were associated with the outcome in Geriatric Units; conversely, among nephrological patients, only female sex were associated with CKD under-reporting.

**TABLE 2:** Factors associated with CKD under-reporting in the study population and single units.

	Overall population		Geriatric units		Nephrology units	
	Age- and sex-adj PR (95% CI)	Fully adj PR (95%CI)	Age- and sex-adj PR (95% CI)	Fully adj PR (95%CI)	Age- and sex-adj PR (95% CI)	Fully adj PR (95%CI)
Age	1.07 (1.06–1.09)	1.05 (1.04–1.07)	1.06 (1.05–1.08)	1.06 (1.04–1.08)	1.00 (0.96–1.04)	1.00 (0.96–1.03)
Female sex	1.25 (1.09–1.43)	1.22 (1.06–1.41)	1.18 (1.03–1.35)	1.19 (1.03–1.37)	2.14 (1.24–3.71)	2.08 (1.20–3.60)
BMI ≥ 25	1.07 (0.89–1.29)	—	1.06 (0.89–1.27)	—	1.75 (0.73–4.19)	—
Number of drugs	0.99 (0.97–1.01)	—	1.04 (1.00–1.07)	—	0.84 (0.69–0.98)	0.77 (0.70–0.90)
Hypertension	1.05 (0.90–1.22)	—	1.17 (0.99–1.38)	—	0.52 (0.28–0.95)	0.57 (0.31–1.06)
CAD	1.11 (0.95–1.30)	—	1.11 (0.95–1.29)	—	0.42 (0.13–1.35)	—
Atrial fibrillation	1.11 (0.98–1.26)	—	1.08 (0.96–1.22)	—	0.92 (0.47–1.81)	—
CHF	1.36 (1.15–1.61)	1.22 (1.05–1.41)	1.28 (1.07–1.53)	1.26 (1.06–1.50)	0.34 (0.11–1.02)	−0.38 (0.13–1.15)
Diabetes	0.97 (0.86–1.10)	—	1.08 (0.94–1.23)	—	0.54 (0.29–0.99)	0.61 (0.33–1.12)
Cerebrovascular disease	0.96 (0.81–1.14)	—	0.91 (0.76–1.08)	—	0.68 (0.21–2.16)	—
Anemia	1.08 (0.95–1.22)	—	1.20 (1.03–1.40)	1.18 (1.02–1.37)	1.21 (0.66–2.23)	—
Geriatric units	4.14 (2.45–6.46)	3.93 (2.53–6.11)	—	—	—	—

### 3.3 Characteristics of drug prescriptions

Overall, the median number of medications prescribed at discharge was seven (5–10), with a prevalence of polypharmacy of 68.1%, slightly higher in nephrology units (72.9%) than in geriatric units (66.3%). The most common drug classes prescribed at discharge (Supplementary Table S1) were proton-pump inhibitors (61.0%), diuretics (59.4%), beta-blockers (50.3%), antithrombotics (47.2%), and RAAS inhibitors (45.8%). The prevalence of inappropriate prescriptions varied widely depending on criteria used: 22 patients (1.1%) according to STOPP criteria, 197 (9.6%) according to Beers criteria, and 290 (14.1%) according to smPC criteria. The overall prevalence of STOPP and/or Beers PIMs and smPC PIMs is reported in Table 3. Patients treated with at least one potentially inappropriate medication according to STOPP and/or Beers criteria at the time of hospital discharge were 210 (10.2%), with higher PIM rates in nephrology (16.2%) vs. geriatrics (8.0%, *p* < 0.001) units. Among patients with CKD, 18.5% met criteria for having at least one STOPP and/or Beers PIM. The distribution of PIM prevalence across eGFR stages underwent a graded increase from G3a to G5, with a steeper increase from G3b to G5 in all settings. The inclusion of smPC criteria for assessment of PIM increased the overall prevalence of patients with at least one cumulative PIM up to 380 (18.5%) of the study population, 174 (11.7%) in Geriatric Units and 206 (37.0%) in Nephrology Units. Among patients with CKD, 24.0% met criteria for having at least one STOPP and/or Beers and/or smPC criteria.

**TABLE 3:** Prevalence of STOPP and/or Beers PIMs and cumulative PIMs in the study population and different settings.

BIS eGFR, *n* (%)*	STOPP and/or Beers PIM	Geriatric STOPP and/or Beers PIMs	Nephrology STOPP and/or Beers PIMs	*p* ^*b*^
≥60	12 (2.5)	10 (2.4)	2 (3.7)	
45–59.9	21 (4.2)	16 (3.8)	5 (6.2)	
30–44.9	57 (9.9)	33 (7.7)	24 (16.3)	
<30	120 (24.5)	61 (28.5)	59 (21.4)	
Total, *n* (%)*	210 (10.2)	120 (8.0)	90 (16.2)	<0.001
*p* ^*a*^	<0.001	<0.001	<0.001	
	All PIM	Geriatric PIMs	Nephrology PIMs	
≥60	18 (3.7)	15 (3.5)	3 (5.6)	
45–59.9	33 (6.5)	22 (5.2)	11 (13.7)	
30–44.9	65 (11.3)	39 (9.1)	26 (17.7)	
<30	264 (54.0)	98 (45.8)	166 (60.4)	
Total, *n* (%)**	380 (18.5)	174 (11.7)	206 (37.0)	<0.001
*p* ^*a*^	<0.001	<0.001	<0.001	

*Percentages were calculated on the total number of patients belonging to each CKD stage; p^*a*^ of the comparisons among prevalence of PIM across CKD stages; p^*b*^ of the comparisons between prevalence of global PIM in nephrology and geriatric units.

**FIGURE 1:**
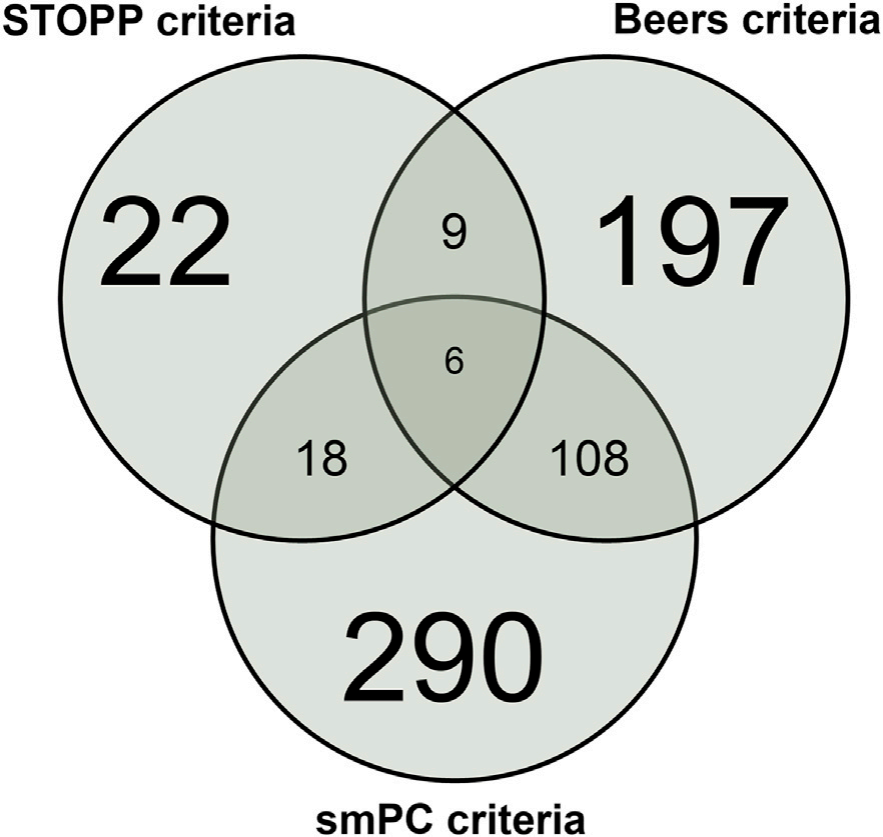
Venn diagram showing the overlap between STOPP, Beers, and smPC PIM in the overall population.

A Venn diagram showing the overlap between the three types of criteria in intercepting patients with at least one PIM is depicted in Figure 1.

A detailed picture of inappropriately prescribed drugs is reported in Supplementary Table S2. Among drugs with a prescription rate of over 10%, PIM rates were higher for fast-intermediate insulins (27.0%) and enoxaparin (8.3%). Among drugs prescribed in 5%–10% of the study population, PIM rates were higher for febuxostat (58.9%), antacids (48.8%), potassium canrenoate (24.2%), spironolactone (21.6%), and metformin (14.4%). Relevant PIMs among less commonly prescribed drugs included ranitidine (52.5%), fondaparinux (46.5%), lercanidipine (38.9%), nebivolol (29.1%), rivaroxaban (27.4%), and duloxetine (26.1%). On the other hand, drugs commonly associated with nephrotoxicity, such as NSAIDs, were prescribed in less than 1% of the study population. Inappropriate prescription of fast-intermediate insulins, enoxaparin, K-sparing diuretics, duloxetine, antacids, rosuvastatin, and alfuzosin was more common in nephrology units.

Factors associated with inappropriate drug use at discharge in the overall study population are shown in Table 4. In brief, the number of drugs, atrial fibrillation, diabetes, and CKD stages were associated with PIMs in the overall study population, independent of criteria used to assess inappropriate prescribing; admission to nephrology units was associated with increased use of at least one cumulative PIM (one STOPP and/or Beers and/or smPC). Furthermore, while the number of drugs and CKD stages were always associated with increased PIM use, diabetes was found to be a risk factor in geriatric units only (Supplementary Tables S3, S4). Despite its relatively high prevalence, under-reporting of CKD in hospital discharge forms was not associated with the outcome in all logistic regression models. After repeating the analysis by using the CKD-EPI equation instead of the BIS equation to estimate the eGFR, we obtained comparable results (Supplementary Tables S5–S8).

**TABLE 4:** Factors associated with PIMs in the overall study population.

	At least one STOPP and/or Beers PIM		At least one cumulative PIM	
	Age- and sex-adj PR (95% CI)	Fully adj PR (95%CI)	Age- and sex-adj PR (95% CI)	Fully adj PR (95%CI)
Age	1.00 (0.99–1.02)	1.00 (0.97–1.02)	0.99 (0.97–1.00)	0.98 (0.96–1.00)
Female sex	0.97 (0.75–1.26)	0.97 (0.72–1.31)	0.97 (0.84–1.13)	1.00 (0.76–1.31)
BMI ≥25	1.18 (0.83–1.68)	—	1.24 (0.86–1.61)	—
Number of drugs	1.14 (1.09–1.20)	1.05 (1.01–1.10)	1.22 (1.16–1.27)	1.14 (1.09–1.19)
Hypertension	1.20 (0.86–1.68)	—	1.33 (1.05–1.69)	1.19 (0.84–1.69)
CAD	0.92 (0.64–1.31)	—	0.94 (0.77–1.15)	—
Atrial fibrillation	1.52 (1.17–1.98)	1.41 (1.03–1.44)	1.22 (1.05–1.42)	1.35 (1.01–1.81)
CHF	1.30 (1.00–1.68)	—	1.15 (1.00–1.32)	—
Diabetes	2.13 (1.56–2.91)	1.79 (1.31–2.45)	1.83 (1.40–2.40)	1.61 (1.21–2.13)
Cerebrovascular disease	0.99 (0.68–1.44)	—	0.95 (0.77–1.19)	—
Anemia	1.70 (1.26–2.30)	1.05 (0.75–1.38)	1.60 (1.22–2.08)	1.05 (0.77–1.41)
Under-reported CKD diagnosis	0.94 (0.71–1.25)	—	0.65 (0.50–0.84)	0.96 (0.68–1.35)
Nephrology setting	2.30 (1.65–3.21)	1.02 (0.71–1.45)	4.69 (3.61–6.09)	1.62 (1.14–2.31)
eGFR stage				
≥60	1	1	1	1
45.59.9	1.69 (0.86–3.31)	1.50 (0.74–3.06)	1.44 (0.98–2.11)	1.51 (0.81–2.81)
30–44.9	3.81 (2.08–6.98)	3.46 (1.85–6.47)	1.82 (1.09–3.01)	2.35 (1.34–4.13)
<30	7.92 (3.64–17.22)	8.66 (4.65–16.12)	2.41 (1.08–5.38)	14.01 (7.36–26.72)

## 4 Discussion

In this study, we investigated the appropriateness of CKD diagnosis and drug use among older patients discharged from geriatric nephrology acute care units. Furthermore, we compared the clinical and pharmacological profiles of patients admitted to geriatric and nephrology wards. Our findings showed that under-reporting of CKD in clinical charts was distinctively prevalent among patients discharged from geriatric wards. The prevalence of inappropriate prescriptions at discharge was up to 18.5% of the study population, with increasing rates as the eGFR decreases and among patients in nephrology units.

### 4.1 Chronic kidney disease underdiagnosis

Identifying patients with CKD during hospital stay and preventing adverse drug events are both recognized as major health issues. Since creatinine values may be misleading and do not accurately estimate renal function in older patients, an array of standardized formulas for estimation of the GFR have been developed and are automatically reported by electronic medical records when a serum creatinine level is obtained (Levey et al., 2009; Schaeffner et al., 2012). Despite these efforts, the current relevant rate of CKD underdiagnosis is reported at the hospital level (Gentile et al., 2009; Tesfaye et al., 2019). Recent data from European (Bosi et al., 2021) and Japanese (Takeuchi et al., 2021) populations revealed that CKD was under-diagnosed in around 23%–25% of the study populations. Consistent with these previous findings, our study results confirmed that under-reporting CKD was relatively common in hospitalized older patients, with up to 50.8% of CKD patients being reported as not having CKD at hospital discharge. Interestingly, the prevalence of under-reporting was much higher in the geriatric setting than in lower but non-negligible rates among patients in uephrology units. Furthermore, age, female sex, atrial fibrillation, CHF, and anemia were associated with CKD under-reporting in geriatric units, while the female sex was the only factor associated with the outcome in Nephrology Units, with a number of drugs showing a negative association. We can speculate that the abovementioned diseases can manifest with more acute and less subtle symptoms, leaving CKD in the background, thereby causing under-reporting. The negative relationship between the number of drugs and CKD under-reporting was evident only in nephrology units and may be due to the increased CKD severity of nephrological patients at an increasing number of drugs, with subsequent increased awareness of CKD diagnosis at discharge. However, as seen in previous studies, the recorded diagnosis of CKD was not associated with drug inappropriateness (Barbieri et al., 2020). In any case, since CKD is set to become the fifth leading global cause of death by 2040 and the second leading cause of death before the end of the century in high-income countries (Wong et al., 2018; Luyckx et al., 2021), it is deemed necessary to improve CKD diagnosis during hospital stay. In this regard, another relevant point is the current debate about the suggested age-dependent CKD definition. Nephrologists are still arguing whether isolated mild eGFR reductions to a level just below 60 ml/min/1.73 m^2^ should even be considered a disease in older adults (Delanaye et al., 2019; Cruz-Jentoft, 2022; Liu et al., 2022). Considering the effect of comorbidity and renal senescence on eGFR decline (Glassock et al., 2020), many authors suggest avoiding the use of a “one-size-fits-all approach” for CKD diagnosis and to rethink CKD definition to include age-specific eGFR thresholds (Delanaye et al., 2019; Glassock et al., 2020). In the case that this approach is accepted, it is clear that clinicians would communicate to patients that stable but mildly reduced renal function (eGFR 45–59 ml/min/1.73 m^2^) is a low-risk situation that can usually be observed with focus on cardiovascular prevention. This consideration might allow a trend to under-recognition of CKD patients in the 3A stage, especially in geriatric settings, where patients are commonly comorbid and at an increased risk of CKD progression, despite the presence of only a mild eGFR decline. In this study, we also found a high prevalence of stage 3A patients, particularly in Geriatric units; so, it is not surprising that the vast majority of CKD under-reporting belonged to stage 3A. In a previous survey from the SIN-SIGG group, we showed that geriatricians were aware of how CKD is relevant to the health of their patients and then to their practice. Indeed, nephrological training is not included in the geriatric curriculum recommended by the Italian Minister of Education (Antonelli Incalzi et al., 2015). The same is also true for the primary care setting. There is then an urgent need to address the unawareness of the CKD concept, starting with the primary care physicians, who are the gatekeepers of the healthcare system, using hospitalization as a second step for recognition. Increased CKD awareness may also result in increased CKD referrals to nephrologists, allowing a detailed treatment of the many and complex factors of comorbidity inherent to CKD at its various stages, not only delaying the start of dialysis for as long as possible but also avoiding the prescription of nephrotoxic drugs.

### 4.2 Prevalence and characteristics of potentially inappropriate medications

The evaluation of drugs prescribed in the study population has revealed a consistently high prevalence of polypharmacy across study settings, with up to 68.1% of all patients being prescribed with at least five medications at discharge; polypharmacy rates were higher in Nephrology Units (72.9%) than in Geriatric Units (66.3%). Polypharmacy burden in CKD was previously described, as older patients with kidney function deterioration often present with multimorbidity that puts them at risk of increased medication use, drug–drug and drug–disease interactions, as well as adverse drug reactions (ADRs) (Roux-Marson et al., 2020). The nephrotoxic medication use among patients with CKD ≥ G3 followed in the outpatient care from Sweden and the US, characterized by two distinct and geographically diverse health care systems, recently emerged as a prominent issue: the prescription of nephrotoxic medications occurred in at least one in five individuals, with non-steroidal anti-inflammatory drugs (NSAIDs) being the most commonly prescribed medications (Bosi et al., 2021); moreover, the CKD-REIN study found an ADR incidence rate of 14.4 per 100 person-years (Laville et al., 2020). Our data are in agreement with those of the previous reports, as patients treated with at least one PIM according to STOPP and Beers criteria at the time of hospital discharge were 10.2% and reached 18.5% when also considering evidence from smPCs; interestingly, prescription of some nephrotoxic drugs, like NSAIDs, was extremely low in the study population. The greater PIM prevalence among patients discharged from nephrology units might be related to the increased CKD severity of patients belonging to this setting. In this regard, recent findings by Tonelli et al. (2018) showed that patients who consulted nephrologists were the most complex to treat because of their more severe renal impairment associated with the common presence of complications, resulting also in a high number of prescribed medications. Moreover, when evaluating PIMs among CKD patients in different clinical settings, previous evidence found that physician primary care providers prescribed the greatest number of PIMs requiring renal adjustment, while the greatest PIM prevalence by specialty occurred among endocrinologists (Tonelli et al., 2018). This may be related to the high need for renal adjustments of many antidiabetic drugs, which were often responsible for the commonest inappropriate prescriptions in our study, along with febuxostat, enoxaparin, and other antithrombotics, K-sparing diuretics, and antacids. Inappropriate use of antidiabetic drugs in CKD may increase the risk of hypoglycemic and cardiovascular events, lactic acidosis, and further progression of kidney dysfunction, without improving glycemic control (By the 2019 American Geriatrics Society Beers Criteria^®^ Update Expert Panel, 2019; Mariano and Biancone, 2021). K-sparing drugs are other well-known causes of PIMs (Tonelli et al., 2018) and are frequently administered to patients with CKD; however, as the eGFR declines, caution is needed for the risk of hyperkalemia and cardiovascular side effects; similarly, use of antithrombotics, such as enoxaparin, direct thrombin inhibitors, and fondaparinux, needs to be tailored to individual renal function in order to decrease the risk of bleeding due to drug accumulation and risk of thrombocytopenia (By the 2019 American Geriatrics Society Beers Criteria^®^ Update Expert Panel, 2019; Brophy and Sica, 2007); another important concern is related to the high inappropriate prescription of antacids, which is confirmed by previous studies and is hard to counteract as many of these drugs are available as over-the-counter medications. Use of these compounds in advanced CKD may lead to surreptitious intake of calcium and magnesium, potentially causing electrolyte disturbances and further eGFR decline (Whittaker et al., 2018a; Stefani et al., 2019). Hence, patients treated with these drugs should be closely monitored in order to reduce/stop treatment when the eGFR is declining over time (Shah et al., 2017; Chen et al., 2019; Stefani et al., 2019). Strategies to increase physician’s awareness of CKD and knowledge of drug nephrotoxicity are needed to reduce prescribing nephrotoxic medications and prevent iatrogenic kidney injury.

### 4.3 Factors associated with potentially inappropriate medications

Despite the existence of several criteria to address drug appropriateness in hospital settings (O’Mahony, 2020; By the 2019 American Geriatrics Society Beers Criteria^®^ Update Expert Panel, 2019), prescription of inappropriate drugs is still a relevant issue in acute care medicine, which needs to be counteracted by several measures. This is particularly bothersome in patients with CKD, who are often multimorbid and taking multiple medications. In this regard, the number of drugs and CKD stages emerged as the two most important factors associated with PIM use in the study population. These results are concordant with those reported in previous studies, showing how polypharmacy, decreased medication adherence, and advanced CKD are risk factors for ADRs (Laville et al., 2020). The association between the number of drugs and PIMs is well-established: several studies showed that increasing the number of medications increases the risk of drug–drug interactions as well as inappropriate prescriptions (Chang et al., 2015; Chahine, 2020; Laville et al., 2020). Similarly, progression through CKD stages was associated with PIM, confirming previous evidence showing how approximately one-third of older adults with CKD staged G3–G5 had at least one PIM, and patients with G4–5 CKD stage shared a 11-fold risk of having a PIM when compared to stage G3 (Silva-Almodóvar et al., 2021). The association between diabetes and PIM risk was not surprising, considering the clinical and pharmacological burden of this disease in CKD patients (Navaneethan et al., 2021).

Overall, we may consider that the incidence of ADRs increases as kidney function deteriorates, as a result of drug-related nephrotoxicity or drug accumulation. In order to avoid iatrogenic injury, the process of drug prescribing needs to take into account aging itself, renal function deterioration, and multimorbidity, including diabetes, hypertension, and heart failure, especially when a wide range of prescribers often do not coordinate treatments. An international European study in patients with advanced CKD showed an overall 90.7% and 42.8% prevalence of polypharmacy and hyperpolypharmacy, respectively, with Italy showing the highest rates across Europe (Hayward et al., 2021). The significant differences in the number of prescribed medications across countries underline the lack of international consensus regarding appropriate prescribing in older patients with CKD. A potential solution to this problem consists of medication reconciliation and approaches based on depression (Whittaker et al., 2018b); the latter, consisting in the systematic process of identifying and discontinuing drugs in the presence of a high harm-to-benefit ratio (Scott et al., 2015), could lead to early interception of nephrotoxic prescriptions in patients with CKD (George et al., 2021; Manski-Nankervis et al., 2021). Key steps in deprescribing include a comprehensive medication review to identify and prioritize PIMs for discontinuation or dose reduction, followed by a plan to withdraw drugs safely and patient monitoring after discontinuation. This process should be integrated with careful and periodic re-evaluation of kidney function in order to tailor drug regimens to eGFR levels (Whittaker et al., 2018b). This will likely improve caution when prescribing medications for patients with CKD and reduce the frequency of adverse health effects related to polypharmacy in this population. Hospital admissions should represent a valuable opportunity to identify CKD and increased CKD awareness, to revise treatment strategies in patients with decreased eGFR, and to define preventive strategies to appropriately manage at-risk patients at discharge.

### 4.4 Strength and limitations

This study has many strengths and limitations. The use of real-world data in older complex patients, who are not generally enrolled in premarketing studies, is a major strength of this study. The multicenter design of the SIN-SIGG study allowed us to include a large number of nephrology and geriatric patients, thus making the study representative of the real-world scenario. Additionally, this is the first study showing a comparative analysis of the prevalence and clinical correlates of PIM use in geriatric and nephrology acute care settings. However, some limitations need to be acknowledged. First, the retrospective design of the study likely represents the main limitation. Thus, our study needs to be prospectively replicated, even involving other countries. Indeed, the lack of data after a patient’s discharge did not allow capturing the relationship between PIM prevalence and the eGFR trajectory over time; moreover, our findings apply to patients hospitalized in acute care units in Italy and cannot be generalized to other settings and/or countries. Additionally, evaluation of kidney function was based only on serum creatinine values, and lack of albuminuria may underestimate CKD prevalence. Furthermore, a lack of data regarding the onset and duration of CKD may have led to underestimation of the real prevalence of CKD.

## 5 Conclusion

In summary, CKD under-reporting and renally inappropriate medication use is common in older patients discharged from acute care units; the high number of PIMs in both nephrology and geriatric settings underline the need to improve appropriate prescribing during hospital stay and to decrease the risk of ADRs and side effects in this highly vulnerable population.

## 6 SIN-SIGG Group Nephrology Units

Filippo Aucella, Giuseppe Gatta, Michele Prencipe, Rachele Grifa [Nephrology and Dialysis Unit, Fondazione “Casa Sollievo della Sofferenza” IRCCS, San Giovanni Rotondo (FG)]; Elena Mancini, Francesca Romani, Roberta Benevento (Nephrology, Dialysis and Hypertension Unit, IRCCS Azienda Ospedaliero-Universitaria di Bologna, Bologna); Gianfranca Cabiddu. (Nephrology and Dialysis Unit, Azienda Ospedaliera Brotzu, Cagliari, Italy); Alessandra Perna, Filippo De Stefano, Carolina Ruosi (Nephrology and Dialysis Unit, Università Vanvitelli, Napoli); Leonardo Calandra, Katia Montalbano, Marco Guarneri (Nephrology and Dialysis Unit, AOU Policlinico, Palermo); Ciro Esposito, Marco Colucci, Giuseppe Sileno, Vittoria Esposito (Nephrology and Dialysis Unit, Istituto Maugeri, Pavia); Francesca Mallamaci, Daniela Leonardis (Nephrology and Dialysis Unit, CNR-IFC, Reggio Calabria); Marcora Mandreoli, Laura Scichilone (Nephrology and Dialysis Unit, Ospedale Santa Maria della Scaletta, Imola); Giorgio Fuiano, Giuseppe Coppolino (Nephrology and Dialysis Unit, Università “Magna Graecia”, Catanzaro).

## 7 SIN-SIGG Group Geriatric Units

Ilaria Lazzari (Department of Geriatric Medicine, Policlinico S. Orsola-Malpighi, Bologna); Alessandra Marengoni (Medicina ad Indirizzo Geriatrico, Spedali Civili Presidio Montichiari, Brescia); Angela Sciacqua, Giuseppe Armentaro (Unit of Geriatric Medicine, Università Magna Graecia, Catanzaro); Andrea Corsonello, Luca Soraci, Ramona Caloiero, Maurizio Leonardo Guerrieri, Annalisa Cozza (Unit of Geriatric Medicine, IRCCS INRCA, Cosenza); Andrea Ungar (Unit of Geriatric Medicine, AOU Careggi, Firenze); Francesco Corica, Salvatore Bonanzinga (Unit of Geriatric Medicine, AOU Policlinico G. Martino, Messina); Giuseppe Bellelli (Acute Geriatric Unit, Ospedale di Monza-ASST Monza, Monza); Dario Leosco (Department of Translational Medical Sciences, Scuola di Specializzazione in Geriatria, Università Vanvitelli, Napoli); Mario Barbagallo (Unit of Geriatric Medicine, AOU Policlinico, Palermo); Fabio Monzani (Geriatric Unit, AOU Pisana, Pisa); Raffaele Antonelli Incalzi, Livio Cortese, Maria Rosaria Pagnotta (Department of Geriatrics, Università Campus Biomedico, Roma); Antonio Greco [Unit of Geriatric Medicine, IRCCS, “Casa Sollievo della Sofferenza”, San Giovanni Rotondo (FG)]; Mario Bo, Vittoria Tibaldi (Section of Geriatrics, Department of Medical Sciences, University of Turin, A.O.U. Città della Salute e della Scienza, Molinette, Torino); Alessandro Cavarape (Clinica Medica, Department of Medicine, University of Udine, Presidio Ospedaliero S. Maria della Misericordia, Udine).

## Data Availability

The raw data supporting the conclusion of this article will be made available by the authors without undue reservation.
