# Supplementary material for: A focus on CKD reporting and inappropriate prescribing among older patients discharged from geriatric and nephrology units throughout Italy: A nationwide multicenter retrospective cross-sectional study
Source: Front Pharmacol. 2022 Oct 14;13:996042. doi: 10.3389/fphar.2022.996042 (PMC9614038; doi:10.3389/fphar.2022.996042)
Supplement: Supplementary file 1 [file DataSheet1.PDF]

Supplementary Box 1. List of criteria used to assess inappropriate prescription at hospital discharge

Criteria for testing inappropriateness	Notes
**STOPP**	1. Digoxin at doses> 125μg / day when eGFR < 30 ml/min/1.73 m^2 (high risk of toxicity if digoxin is not monitored).<br>2. Direct thrombin inhibitors (eg dabigatran) if eGFR < 30 ml/min/1.73 m^2 (bleeding risk).<br>3. Factor Xa inhibitors (eg rivaroxaban, apixaban) if eGFR < 15 ml/min/1.73 m^2 (bleeding risk).<br>4. NSAIDs if eGFR < 50 ml/min/1.73 m^2 (risk of deterioration of renal function).<br>5. Colchicine if eGFR < 10 ml ml/min/1.73 m^2 (risk of toxicity).<br>6. Metformin if eGFR < 30 ml/min/1.73 m^2 (risk of lactic acidosis).
**Beers**	1) Ciprofloxacin at unadjusted dose when eGFR < 30 ml/min/1.73 m^2.<br>2) Trimethoprim/sulphametoxazole at unadjusted dose when eGFR 15-30 ml/min/1.73 m^2, or at any dose when eGFR < 15 ml/min/1.73 m^2.<br>3) Fast-intermediate insulins with no basal insulin when eGFR < 30 ml/min/1.73 m^2.<br>4) Metformin when eGFR < 30 ml/min/1.73 m^2.<br>5) Amiloride when eGFR < 30 ml/min/1.73 m^2.<br>6) Apixaban when eGFR < 25 ml/min/1.73 m^2.<br>7) Dabigatran when eGFR < 30 ml/min/1.73 m^2.<br>8) Edoxaban at unadjusted dose when eGFR 15-50 ml/min/1.73 m^2, or at any dose when eGFR < 15 ml/min/1.73 m^2 or > 95 ml/min/1.73 m^2.<br>9) Fondaparinux when eGFR < 30 ml/min/1.73 m^2.<br>10) Enoxaparin at unadjusted dose when eGFR 15-30 ml/min/1.73 m^2 or at any dose when eGFR < 15 ml/min/1.73 m^2.<br>11) Rivaroxaban at unadjusted dose when eGFR 15-50 ml/min/1.73 m^2 or at any dose when eGFR < 15 ml/min/1.73 m^2 in patients diagnosed with non-valvular atrial fibrillation.<br>12) Spironolactone when eGFR < 30 ml/min/1.73 m^2.<br>13) Triamterene when eGFR < 30 ml/min/1.73 m^2.<br>14) Duloxetine when eGFR < 30 ml/min/1.73 m^2.<br>15) Gabapentin at unadjusted dose when eGFR < 60 ml/min/1.73 m^2.<br>16) Levetiracetam at unadjusted dose when eGFR < 80 ml/min/1.73 m^2.<br>17) Pregabalin at unadjusted dose when eGFR < 60 ml/min/1.73 m^2.<br>18) Cimetidine at unadjusted dose when eGFR < 50 ml/min/1.73 m^2.<br>19) Nizatine at unadjusted dose when eGFR < 50 ml/min/1.73 m^2.<br>20) Famotidine at unadjusted dose when eGFR < 50 ml/min/1.73 m^2.<br>21) Ranitidine at unadjusted dose when eGFR < 50 ml/min/1.73 m^2.<br>22) Probenecid when eGFR < 30 ml/min/1.73 m^2.<br>23) Colchicine when eGFR < 30 ml/min/1.73 m^2.<br>24) Dual RAS ihibitors when eGFR < 45 ml/min/1.73 m^2
**smPCs**	1. Antacids when eGFR < 30 ml/min/1.73 m^2.

2. Trimethoprim/sulfametoxazole when eGFR < 30 ml/min/1.73 m^2.
3. Cetirizine when eGFR < 10 ml/min/1.73 m^2.
4. NSAIDs when eGFR < 30 ml/min/1.73 m^2.
5. Dual RAS inhibitors when eGFR < 60 ml/min/1.73 m^2
6. Dabigatran when eGFR < 30 ml/min/1.73 m^2.
7. Fondaparinux when eGFR < 20 ml/min/1.73 m^2.
8. Beta-blockers:
    a. Nebivolol when eGFR < 30 ml/min/1.73 m^2.
    b. Sotalol when eGFR < 10 ml/min/1.73 m^2.
9. Lercanidipine when eGFR < 30 ml/min/1.73 m^2.
10. Potassium-sparing diuretics:
    a. Canrenone when eGFR < 30 ml/min/1.73 m^2.
    b. Eplerenone when eGFR < 30 ml/min/1.73 m^2.
    c. Hydrochlorothiazide when eGFR < 30 ml/min/1.73 m^2.
    d. Indapamide when eGFR < 30 ml/min/1.73 m^2.
    e. Potassium canreonate when eGFR < 30 ml/min/1.73 m^2.
    f. Spironolactone when eGFR < 30 ml/min/1.73 m^2.
11. Drugs for osteoporosis:
    a. Alendronic acid when eGFR < 30 ml/min/1.73 m^2.
    b. Risedronic acid when eGFR < 35 ml/min/1.73 m^2.
    c. Zoledronic acid when eGFR < 35 ml/min/1.73 m^2.
12. Antidiabetics:
    a. Acarbose when eGFR < 15 ml/min/1.73 m^2.
    b. Glibenclamide when eGFR < 30 ml/min/1.73 m^2.
    c. Gliclazide when eGFR < 30 ml/min/1.73 m^2.
    d. Glimepiride when eGFR < 30 ml/min/1.73 m^2.
    e. Metformin at unadjusted dose when eGFR < 60 ml/min/1.73 m^2.
    f. Sitagliptin at unadjusted dose when eGFR < 45 ml/min/1.73 m^2.
    g. Fast-intermediate insulins with no basal or long-acting insulin.
13. Lipid modifying agents:
    a. Fenofibrate when eGFR < 30 ml/min/1.73 m^2.
    b. Gemfibrozil when eGFR < 30 ml/min/1.73 m^2.
    c. Rosuvastatin when eGFR < 30 ml/min/1.73 m^2.
14. Anti-gout medications:
    a. Colchicine when eGFR < 30 ml/min/1.73 m^2.
    b. Febuxostat at unadjusted dose when eGFR < 30 ml/min/1.73 m^2.
    c. Allopurinol at unadjusted dose when eGFR < 30 ml/min/1.73 m^2.
15. Duloxetine when eGFR < 30 ml/min/1.73 m^2.
16. Clozapine when eGFR < 30 ml/min/1.73 m^2.
17. Danazol when eGFR < 30 ml/min/1.73 m^2.
18. Raloxifene when eGFR < 30 ml/min/1.73 m^2.
19. Alfuzosin when eGFR < 30 ml/min/1.73 m^2.
20. Edoxaban at unadjusted dose when eGFR 15-50 ml/min/1.73 m^2 or at any dose when eGFR < 15 ml/min/1.73 m^2

Supplementary Table 1. Most common medications prescribed at discharge in the overall population and in Geriatric and Nephrology Units

	Overall population (n=2,057)	Geriatric Units (n=1,497)	Nephrology Units, (n=560)	*p*
**PPI**, n (%)	1,254 (61.0)	908 (60.6)	346 (61.8)	0.676
**Antidiabetics**, n (%)	551 (26.8)	384 (25.6)	167 (29.8)	0.065
Insulins, n (%)	304 (14.8)	205 (13.7)	99 (17.7)	0.028
Metformin, n (%)	160 (7.8)	138 (9.2)	22 (3.9)	<0.001
Sulphan/repagl. , n (%)	111 (5.4)	72 (4.8)	39 (7.0)	0.069
DPP4-i, n (%)	106 (5.1)	72 (4.8)	34 (6.1)	0.298
**Antithrombotics**, n (%)	970 (47.2)	740 (49.4)	230 (41.1)	<0.001
Vitamin K antagonists, n (%)	203 (9.9)	125 (8.3)	78 (13.9)	<0.001
Heparin, n (%)	248 (12.1)	179 (12.0)	69 (12.3)	0.881
Antiplatelet, n (%)	303 (15.0)	219 (14.6)	84 (15.0)	0.888
DOACs, n (%)	253 (12.3)	232 (15.5)	21 (3.7)	<0.001
**Antianemics**, n (%)	398 (19.3)	184 (12.3)	214 (38.2)	<0.001
Iron, n (%)	119 (5.8)	66 (4.4)	53 (9.5)	<0.001
Vitamin B12 and folic acid, n (%)	114 (5.5)	81 (5.4)	33 (5.9)	0.751
ESA, n (%)	235 (11.4)	64 (42.7)	171 (30.5)	<0.001
**Antiarrhythmics**, n (%)	168 (8.2)	133 (8.9)	35 (6.3)	0.064
Digoxin, n (%)	57 (2.8)	50 (3.3)	7 (1.2)	0.016
**Diuretics**, n (%)	1,222 (59.4)	914 (61.1)	308 (55.0)	0.015
Loop diuretics, n (%)	1,106 (53.8)	821 (54.8)	285 (50.9)	0.121
Potassium-sparing diuretics	306 (14.9)	237 (15.8)	69 (12.3)	0.054
Thiazides, n (%)	44 (2.1)	42 (2.8)	2 (0.4)	0.001
**Beta-blockers**, n (%)	1,034 (50.3)	757 (50.6)	277 (49.5)	0.692
**Calcium-channel blockers**, n (%)	449 (21.8)	273 (18.2)	176 (31.4)	<0.001
**RAS inhibitors**, n (%)	945 (45.8)	690 (46.1)	255 (45.5)	0.860
**Lipid-modifying agents**, n (%)	635 (30.9)	407 (27.2)	228 (40.7)	<0.001
Statins, n (%)	610 (29.6)	395 (26.4)	215 (38.4)	<0.001
Ezetimibe, n (%)	74 (3.6)	28 (1.9)	46 (8.2)	<0.001
**NSAIDs, n (%)**	17 (0.8)	11 (0.7)	6 (1.1)	0.633
**Paracetamol, n (%)**	75 (3.6)	57 (3.8)	18 (3.2)	0.612
**Opioids, n (%)**	74 (3.6)	61 (4.1)	13 (2.3)	0.077
**Anticonvulsants, n(%)**	203 (9.9)	151 (10.1)	52 (9.3)	0.646
**Psycholeptics, n (%)**	409 (19.9)	314 (21.0)	95 (17.0)	0.049
**Anti-gout agents, n (%)**	557 (27.1)	293 (19.6)	264 (47.1)	<0.001

Supplementary Table 2. Description of Potentially Inappropriate Medications (PIMs) in the study population.

Potentially inappropriate medication	Overall use	Reference	PIM criteria	PIM use	PIM in Geriatric Units	PIM in Nephrology Units	*p*
Allopurinol	406 (19.6)	smPC	Avoid when eGFR < 30 ml/min/1.73 m^2	17/406 (4.2)	3/237 (1.3)	14/169 (8.3)	<0.001
Fast-intermediate insulins	233 (11.3)	Beers, smPC	Avoid when not associated with basal or long-acting insulin	63/233 (27.0)	36/150 (24.0)	27/83 (32.5)	0.007
Enoxaparin	216 (10.5)	Beers, smPC	Adjust dose when eGFR 15-30 ml/min/1.73 m^2 Avoid when eGFR < 15 ml/min/1.73 m^2	18/216 (8.3)	13/164 (7.9)	5/52 (9.6)	0.993
Factor Xa inhibitors	206 (10.0)	STOPP	Avoid when eGFR < 15 ml/min/1.73m^2	1/207 (0.5)	1/187 (0.5)	0/20 (0)	0.743
Potassium canreonate	178 (8.6)	smPC	Avoid when eGFR < 30 ml/min/1.73 m^2	43/178 (24.2)	28/148 (18.9)	15/30 (50.0)	<0.001
Metformin	160 (7.8)	STOPP/Beers	Avoid when eGFR < 30 ml/min/1.73 m^2	6/160 (3.8)	6/138 (4.3)	0/22 (0)	0.319
		smPC	Adjust dose when eGFR < 60 ml/min/1.73m^2	23/160 (14.4)	19/138 (13.8)	4/22 (18.2)	0.401
Febuxostat	151 (7.3)	smPC	Avoid when eGFR < 30 ml/min/1.73 m^2	89/151 (58.9)	21/56 (37.5)	68/95 (71.6)	<0.001
		smPC	Adjust dose when eGFR 30-60 ml/min/1.73m^2	23/160 (14.4)	19/138 (13.8)	4/22 (18.2)	0.407
Spironolactone	116 (5.6)	Beers, smPC	Avoid when eGFR < 30 ml/min/1.73 m^2	25/116 (21.6)	11/82 (13.4)	14/34 (41.2)	0.001
Antacids	125 (6.1)	smPC	Avoid when eGFR < 30 ml/min/1.73 m^2	61/125 (48.8)	12/67 (17.9)	49/58 (84.5)	<0.001

Apixaban	95 (4.6)	Beers	eGFR < 25 ml/min/1.73m^2	4/95 (4.2)	3/87 (3.4)	1/8 (12.5)	0.222
Rivaroxaban	84 (4.1)	Beers, smPC	Adjust dose when eGFR 15-50 ml/min/1.73 m^2 Avoid when eGFR < 15 ml/min in presence of non-valvular atrial fibrillation Avoid when eGFR < 30 ml/min in prophylaxis of VTE	23/84 (27.4)	17/74 (23.0)	6/10 (60.0)	<0.001
Ranitidine	80 (3.9)	Beers	Adjust dose when eGFR < 50 ml/min/1.73 m^2	42/80 (52.5)	19/50 (38.0)	23/30 (76.7)	<0.001
Pregabalin	70 (3.4)	Beers	Adjust dose when eGFR < 60 ml/min/1.73 m^2	0/70 (0)	-	-	-
Rosuvastatin	59 (2.9)	smPC	Avoid when eGFR < 30 ml/min/1.73 m^2	11/59 (18.6)	1/39 (2.6)	10/20 (50.0)	<0.001
Digoxin	57 (2.8)	STOPP	Adjust dose when eGFR < 30 ml/min/1.73 m^2	2/7 (3.5)	1/50 (2.0)	1/7 (14.3)	0.918
Nebivolol	55 (2.7)	smPC	Avoid when eGFR < 30 ml/min/1.73 m^2	16/55 (29.1)	2/33 (6.1)	14/22 (63.6)	<0.001
Hydrochlorothiazide	51 (2.5)	smPC	Avoid when eGFR < 30 ml/min/1.73 m^2	2/51 (3.9)	0/45 (0)	2/6 (33.3)	0.123
Alfuzosin	47 (2.3)	smPC	Avoid when eGFR < 30 ml/min/1.73 m^2	11/47 (23.4)	5/33 (15.1)	6/14 (42.9)	<0.001
Direct thrombin inhibitors	46 (2.2)	STOPP	Avoid when eGFR < 30 ml/min/1.73 m^2	3/46 (6.5)	3/45 (6.7)	0/1 (0)	0.789
Levetiracetam	44 (2.1)	Beers	Adjust dose when eGFR < 80 ml/min/1.73 m^2	0	-	-	-
Sitagliptin	41 (2.0)	smPC	Adjust dose when eGFR < 45 ml/min/1.73 m^2	15/41 (36.6)	10/34 (29.4)	5/7 (71.4)	<0.001
Lercanidipine	36 (1.7)	smPC	eGFR < 30 ml/min/1.73 m^2	14/36 (38.9)	2/20 (10.0)	12/16 (75.0)	<0.001
Edoxaban	28 (1.4)	smPC	Adjust dose when eGFR 15-50 ml/min/1.73 m^2	12/28 (42.8)	10/26 (38.5)	2/2 (100.0)	<0.001

			Avoid when eGFR < 15 ml/min/1.73 m^2				
Gabapentin	28 (1.4)	Beers	Adjust dose when eGFR < 60 ml/min/1.73 m^2	0/28 (0)	-	-	-
Duloxetine	23 (1.1)	Beers, smPC	Avoid when eGFR < 30 ml/min/1.73 m^2	6/23 (26.1)	3/20 (15.0)5	3/3 (100.0)	0.002
Fondaparinux	22 (1.1)	Beers, smPC	Avoid when eGFR < 30 ml/min/1.73 m^2	10/22 (45.5)	9/21 (42.9)	1/1 (100.0)	0.262
Acarbose	20 (1.0)	smPC	Avoid when eGFR < 25 ml/min/1.73 m^2	2/20 (10.0)	0/9 (0)	2/11 (18.2)	0.129
Dual RAS inhibitors	18 (0.9)	Beers	Avoid when eGFR < 45 ml/min/1.73 m^2	10/18 (55.6)	3/4 (75.0)	7/14 (50.0)	0.375
		smPC	Avoid when eGFR < 60 ml/min/1.73 m^2	16/18 (88.9)	4/4 (100.0)	12/14 (85.7)	<0.001
Ciprofloxacin	17 (0.8)	Beers, smPC	Adjust dose when eGFR < 30 ml/min/1.73 m^2	0/17	-	-	-
NSAIDs	17 (0.8)	STOPP, smPC	Avoid when eGFR < 50 ml/min/1.73 m^2	10/17 (58.8)	4/11 (36.4)	6/6 (100.0)	<0.001
Trimethoprim / sulphometoxazole	15 (0.8)	Beers, smPC	Adjust dose when eGFR 15-30 ml/min/1.73 m^2; contraindicated when eGFR < 15 ml/min/1.73 m^2	2/15 (13.3)	0/5 (0)	2/10 (20.0)	0.881
Dabigatran	13 (0.6)	Beers, smPC	Avoid when eGFR < 30 ml/min/1.73 m^2	1/13 (7.7)	1/12 (8.3)	0/1 (0)	0.999
Glimepiride	9 (0.4)	smPC	Avoid when eGFR < 30 ml/min/1.73 m^2	1/9 (11.1)	1/6 (16.7)	0/3 (0)	0.999
Indapamide	8 (0.4)	smPC	Avoid when eGFR < 30 ml/min/1.73 m^2	1/8 (12.5)	1/8 (12.5)	0/0	0.998
Alendronic acid	7 (0.3)	smPC	Avoid when eGFR < 30 ml/min/1.73 m^2	2/7 (28.6)	2/7 (28.6)	0/0	0.943
Colchicine	7 (0.3)	Beers, smPC	Adjust dose when eGFR < 30 ml/min/1.73 m^2	0/7	-	-	-

Risedronic acid	6 (0.3)	smPC	Avoid when eGFR < 35 ml/min/1.73 m^2	1/6 (16.7)	1/6 (16.7)	0	0.999
Fenofibrate	5 (0.2)	smPC	Avoid when eGFR < 30 ml/min/1.73 m^2	4/5 (80.0)	2/2 (100.0)	2/3 (66.7)	<0.001
Eplerenone	4 (0.2)	smPC	Avoid when eGFR < 30 ml/min/1.73 m^2	1/4 (25.0)	1/3 (33.3)	0/1	0.943
Glibenclamide	1 (0.05)	smPC	Avoid when eGFR < 30 ml/min/1.73 m^2	0/1 (0)	-	-	-
Canrenone	1 (0.05)	smPC	Avoid when eGFR < 30 ml/min/1.73 m^2	0/1 (0)	-	-	-
Zoledronic acid	1 (0.05)	smPC	Avoid when eGFR < 35 ml/min/1.73 m^2	0/1 (0)	-	-	-
Glibenclamide	1 (0.05)	smPC	Avoid when eGFR < 30 ml/min/1.73 m^2	0/1 (0)	-	-	-

Note: Drugs with a prescription rate of 0 % in the study population were not reported.

Supplementary Table 3. **Factors associated with PIMs in geriatric Units**

	At least 1 STOPP-Beers PIM PR (95%CI)		At least 1 Cumulative PIM PR (95%CI)	
	**Age- and sex-adj PR (95% CI)**	**Fully adj PR (95%CI)**	**Age- and sex-adj PR (95%CI)**	**Fully adj PR (95%CI)**
Age	1.01 (0.99-1.04)	0.98 (0.95-1.01)	1.01 (0.99-1.03)	0.96 (0.94-0.99)
Female sex	1.10 (0.81-1.71)	1.11 (0.72-1.63)	1.17 (0.91-1.23)	1.27 (0.87-1.84)
BMI ≥ 25	1.03 (0.84-1.33)	-	1.03 (0.87-1.18)	-
Number of drugs	1.09 (1.03-1.19)	1.03 (0.85-1.09)	1.17 (1.12-1.23)	1.09 (1.02-1.16)
Hypertension	1.14 (0.773-1.84)	-	1.35 (0.91-2.06)	-
CAD	0.95 (0.53-1.44)	-	0.96 (0.61-1.45)	-
Atrial fibrillation	1.63 (1.18-2.53)	1.32 (0.891-2.08)	1.89 (1.36-2.61)	1.39 (0.95-2.05)
CHF	1.57 (1.08-2.29)	0.91 (0.59-1.42)	1.79 (1.30-2.46)	0.93 (0.63-1.38)
Diabetes	2.77 (1.88-4.08)	2.52 (1.77-3.11)	2.42 (1.75-3.37)	2.10 (1.42-3.13)
Cerebrovascular disease	1.13 (0.65-1.82)	-	0.89 (0.55-1.37)	-
Anemia	1.47 (1.00-2.21)	-	1.71 (1.22-2.42)	1.02 (0.69-1.52)
Under-reported CKD diagnosis	1.33 (0.92-1.97)	-	1.14 (0.82-1.59)	-
eGFR stage				
≥ 60	1	1	1	1
45.59.9	1.84 (0.83-4.28)	1.62 (0.75-3.19)	1.80 (0.92-3.62)	1.46 (0.74-2.97)
30-44.9	4.25 (2.10-9.40)	3.51 (1.56-7.65)	3.73 (2.01-7.26)	2.68 (1.41-5.32)
<30	11.12 (7.86-16.56)	9.41 (5.67-12.45)	34.60 (18.90-67.33)	26.66 (14.20-53.07)

Supplementary Table 4. **Factors associated with PIMs in Nephrology Units**

	At least 1 STOPP or Beers PIM		At least 1 STOPP or Beers or smPC PIM	
	**Age- and sex-adj PR (95%CI)**	**Fully adj PR (95%CI)**	**Age- and sex-adj PR (95%CI)**	**Fully adj PR (95%CI)**
Age	1.03 (1.01-1.05)	1.02 (0.99-1.05)	1.03 (1.00-1.05)	1.01 (0.98-1.04)
Female sex	0.84 (0.52-1.33)	0.88 (0.54-1.42)	0.80 (0.56-1.15)	0.73 (0.47-1.12)
BMI ≥ 25	0.94 (0.69-1.27)	-	1.11 (0.88-1.41)	-
Number of drugs	1.14 (1.07-1.22)	1.10 (1.03-1.15)	1.27 (1.20-1.35)	1.21 (1.14-1.30)
Hypertension	1.19 (0.66-2.28)	-	1.49 (0.94-2.43)	-
CAD	0.95 (0.47-1.76)	-	1.02 (0.62-1.66)	-

Atrial fibrillation	1.66 (0.98-2.78)	-	1.42 (0.93-2.17)	-
CHF	1.54 (0.87-2.66)	-	1.71 (1.09-2.70)	1.08 (0.63-1.84)
Diabetes	1.69 (1.07-2.67)	1.24 (0.817-1.56)	1.96 (1.38-2.80)	1.28 (0.83-1.94)
Cerebrovascular disease	0.95 (0.40-2.01)	-	1.65 (0.90-3.01)	-
Anemia	1.97 (1.13-3.63)	1.41 (0.78-2.61)	2.08 (1.38-3.17)	1.03 (0.62-1.69)
Under-reported CKD diagnosis	1.32 (0.60-2.68)	-	0.62 (0.32-1.17)	-
eGFR stage				
≥ 60	1	1	1	1
45.59.9	1.69 (0.75-12.11)	1.47 (0.66-1.62)	2.59 (0.76-11.90)	2.08 (0.59-9.80)
30-44.9	4.80 (1.35-3.59)	3.74 (1.03-24.06)	3.46 (1.15-15.02)	2.34 (0.75-10.34)
<30	6.67 (1.97-41.54)	4.45 (1.28-28.13)	25.26 (8.95-105.97)	16.43 (5.62-70.33)

Supplementary Table 5. Prevalence of CKD stages and misdiagnosis when using CKD-EPI equation to estimate eGFR in the overall study population and the two distinct settings.

	Overall population (n=2,057)	Geriatric Units (n=1,497)	Nephrology Units, (n=560)	*p*
eGFR, ml/min/m^2, median(IQR)	49.2 (30.0-72.8)	56.6 (36.7-77.1)	28.3 (18.1-48.2)	<0.001
CKD-EPI eGFR, ml/min/m^2, n (%)				<0.001
≥ 60	776 (37.7)	609 (40.7)	54 (9.6)	
45-59.9	370 (18.0)	288 (19.2)	46 (8.2)	
30-44.9	395 (19.2)	315 (21.0)	100 (17.9)	
≤30	516 (25.1)	285 (19.0)	360 (64.3)	
CKD diagnosis, n (%)	1,194 (58.0)	726 (48.5)	468 (83.6)	<0.001
Prevalence of under-reported diagnosis of CKD at discharge	511 (42.8)	470 (64.7)	41 (8.8)	<0.001

Supplementary Table 6. Factors associated with CKD under-reporting in the overall study population and distinct settings, when using CKD-EPI to estimate GFR.

	Overall population		Geriatric Units		Nephrology Units	
	Age- and sex-adj PR (95%CI)	Fully adj PR (95%CI)	Age- and sex-adj PR (95%CI)	Fully adj PR (95%CI)	Age- and sex-adj PR (95%CI)	Fully adj PR (95%CI)
Age	1.05 (1.04-1.07)	1.03 (1.01-1.04)	1.04 (1.03-1.06)	1.04 (1.02-1.05)	0.99 (0.94-1.03)	0.98 (0.94-1.03)
Female sex	1.56 (1.27-1.93)	1.65 (1.32-2.05)	1.52 (1.22-1.91)	1.66 (1.31-2.11)	1.93 (1.01-3.75)	1.79 (0.90-3.59)
BMI ≥ 25	1.01 (0.89-1.15)	-	0.91 (0.80-1.04)	-	1.70 (1.07-2.75)	1.80 (1.12-2.97)

Number of drugs	1.02 (0.98-1.05)	-	1.09 (1.05-1.13)	1.04 (1.01-1.09	0.78 (0.70-0.87)	0.78 (0.69-0.87)
Hypertension	0.93 (0.73-1.20)	-	1.06 (0.82-1.39)	-	0.55 (0.27-1.20)	-
CAD	1.30 (0.99-1.70)	-	1.35 (1.01-1.80)	1.05 (0.77-1.43)	0.45 (0.13-1.20)	-
Atrial fibrillation	1.48 (1.19-1.84)	1.20 (0.94-1.51)	1.51 (1.19-1.90)	1.21 (0.94-1.54)	0.86 (0.35-1.89)	-
CHF	1.96 (1.58-2.42)	1.58 (1.25-1.99)	1.92 (1.53-2.41)	1.62 (1.26-2.08)	0.45 (0.13-1.20)	-
Diabetes	1.03 (0.82-1.28)	-	1.22 (0.96-1.56)	-	0.55 (0.26-1.08)	-
Cerebrovascular disease	1.03 (0.77-1.38)	-	0.99 (0.73-1.34)	-	0.26 (0.01-1.28)	-
Anemia	1.72 (1.38-2.15)	1.98 (1.57-2.50)	2.06 (1.63-2.61)	1.93 (1.52-2.45)	2.21 (1.00-5.59)	-
Geriatric Units	4.90 (3.52-6.98)	5.14 (3.66-7.39)	-	-	-	-

Supplementary Table 7. Prevalence of STOPP and Beers PIMs and cumulative PIMs in the study population and different settings when using CKD-EPI equation to estimate GFR.

	**STOPP or Beers PIMs**	**Geriatric STOPP or Beers PIMs**	**Nephrology STOPP or Beers PIMs**	p^b
≥60	15 (1.9)	12 (1.7)	3 (3.6)	
45-59.9	17 (4.5)	12 (4.1)	5 (6.7)	
30-44.9	42 (10.6)	23 (7.9)	19 (18.3)	
<30	126 (24.8)	62 (28.8)	64 (21.8)	
**Total, n (%) ****	**200 (9.7)**	**109 (7.3)**	**91 (16.4)**	**<0.001**
p^a	<0.001	<0.001	<0.001	
	**STOPP or Beers or smPC PIMs**	**Geriatric STOPP or Beers or smPC PIMs**	**Nephrology STOPP or Beers or smPC PIMs**	p^b
≥60	24 (3.1)	20 (2.9)	4 (4.8)	
45-59.9	23 (6.2)	15 (5.1)	8 (10.7)	
30-44.9	45 (11.4)	26 (8.9)	19 (18.3)	
<30	284 (55.8)	103 (47.9)	181 (61.6)	
**Total, n (%) ****	**376 (18.3)**	**164 (11.0)**	**212 (38.1)**	**<0.001**
p^a	<0.001	<0.001	<0.001	

Supplementary Table 8. Factors associated with PIMs in the overall study population when using CKD-EPI equation to estimate GFR

	**At least 1 STOPP-Beers PIM**	**At least 1 Stopp, Beers and smPC PIM**

	**Age- and sex-adj PR (95%CI)**	**Fully adj PR (95%CI)**	**Age- and sex-adj PR (95%CI)**	**Fully adj PR (95%CI)**
Age	0.99 (0.98-1.01)	0.99 (0.97-1.02)	0.98 (0.96-1.00)	0.98 (0.96-1.00)
Female sex	0.99 (0.73-1.33)	1.00 (0.73-1.38)	0.91 (0.73-1.15)	0.98 (0.73-1.31)
BMI ≥ 25	0.93 (0.78-1.12)	-	0.97 (0.84-1.12)	-
Number of drugs	1.16 (1.11-1.22)	1.06 (1.01-1.11)	1.25 (1.20-1.30)	1.16 (1.10-1.21)
Hypertension	1.19 (0.83-1.74)	-	1.52 (1.13-2.06)	1.16 (0.89-1.69)
CAD	0.90 (0.59-1.33)	-	0.96 (0.70-1.29)	-
Atrial fibrillation	1.61 (1.18-2.19)	1.41 (0.99-2.00)	1.41 (1.10-1.80)	1.30 (0.94-1.79)
CHF	1.43 (1.05-1.93)	1.03 (0.72-1.46)	1.28 (1.01-1.63)	0.95 (0.69-1.32)
Diabetes	2.51 (1.86-3.40)	1.90 (1.37-2.64)	2.32 (1.84-2.92)	1.68 (1.24-2.26)
Cerebrovascular disease	1.03 (0.66-1.55)	-	0.99 (0.71-1.37)	-
Anemia	1.94 (1.40-2.72)	1.03 (0.72-1.48)	2.35 (1.82-3.06)	1.06 (0.76-1.46)
Under-reported CKD diagnosis	1.57 (1.14-2.17)	1.30 (0.86-1.97)	1.02 (0.78-1.33)	0.98 (0.67-1.44)
Nephrology Units	2.63 (1.92-3.59)	1.14 (0.74-1.76)	5.12 (4.00-6.57)	1.52 (1.04-2.21)
eGFR stage				
≥ 60	1	1	1	1
45.59.9	2.49 (1.23-5.11)	1.95 (0.91-4.18)	2.24 (1.24-4.04)	1.77 (0.94-3.33)
30-44.9	6.20 (3.46-11.70)	4.47 (2.34-8.89)	4.45 (2.69-7.56)	3.06 (1.75-5.44)
<30	16.92 (10.08-30.51)	12.03 (6.57-23.26)	43.27 (28.20-69.34)	102.50 (56.74-202.40)

***Number of drugs net of those associated with STOPP and Beers PIMs or Number of drugs net of those associated with STOPP, Beers and smPC PIM**
